# Experimental Studies on 3D Printing of Automatically Designed Customized Wrist-Hand Orthoses

**DOI:** 10.3390/ma13184091

**Published:** 2020-09-15

**Authors:** Filip Górski, Radosław Wichniarek, Wiesław Kuczko, Magdalena Żukowska, Monika Lulkiewicz, Przemysław Zawadzki

**Affiliations:** Faculty of Mechanical Engineering, Poznan University of Technology, Piotrowo 3 STR, 61-138 Poznan, Poland; radoslaw.wichniarek@put.poznan.pl (R.W.); wieslaw.kuczko@put.poznan.pl (W.K.); magdalena.k.zukowska@doctorate.put.poznan.pl (M.Ż.); monika.lulkiewicz@student.put.poznan.pl (M.L.); przemyslaw.zawadzki@put.poznan.pl (P.Z.)

**Keywords:** additive manufacturing, mechanical properties, medical 3D printing, orthopedic supplies, personalization

## Abstract

The paper presents results of research conducted on a batch of additively manufactured individualized openwork wrist–hand orthoses made of thermoplastics and designed automatically based on 3D-scanned geometry of a given patient. The aim of the work was to establish an automated design process and find a reliable set of parameters for rapid and affordable manufacturing of usable orthoses on popular 3D printers, with little or no supervision of the process. The paper presents motivations, methodology of automated design, plan of manufacturing and testing, the obtained results in terms of process stability, fit and assessment by patient and strength of the obtained orthoses. Almost 100 manufacturing processes of ready-to-use orthosis parts were carried out in a controlled environment and their results were analyzed thoroughly. The results are promising, as most of the obtained products fulfil the strength criteria, although not all of them meet the economic criteria. As a result, a recommended set of process parameters was determined. These parameters were included in a prototype of the automated design and in a production system developed by the authors.

## 1. Introduction

Rapid development of additive manufacturing technologies, also known as layered manufacturing technologies (or in recent years as 3D printing) has significantly decreased the time needed for implementation of a new product. Additive manufacturing processes make it possible to obtain physical, 3D shapes of nearly any complexity, directly from the digital representation of a product (usually a model made in a Computer Aided Design – CAD system) [1]. There is no need to use any specialized tooling besides the equipment of the manufacturing machine. These technologies are invaluable when there is a need of quick manufacturing of a physical prototype of a designed part [1], which is especially crucial in medicine [2,3,4]. The 3D printing processes can also be useful in the field of foods and nutrition [5], patient education [6] and teaching of resident physicians [7].

A wide spectrum of additive manufacturing varieties makes it possible to manufacture products from many types of materials [8,9]. However, in relation to traditional technologies (casting, machining and plastics molding), additive manufacturing has significant constraints related to the efficiency, quality, and above all, physical and chemical properties of manufactured products [10]. Therefore, most users of additive manufacturing in industry continue to use the technology for prototypes, although as of 2019, the production of ready-to-use orthosis parts is much higher than earlier [11]. There are also many uncertainties in applying the technology in medicine, for direct use by patients, as most available designs tend to be physical prototypes with limited or no clinical input or validation [12].

One of the most commonly used additive manufacturing technologies for industrial purposes is fused-deposition modeling (FDM; alternatively known as fused-filament fabrication), which can be used to obtain parts out of thermoplastic materials. FDM is so popular, that often in media reports the general “3D Printing” term is incorrectly used to describe it. The most widespread build materials are acrylonitrile butadiene styrene (ABS) and polylactic acid (PLA), which ensure relatively good strength and acceptable thermal shrinkage, and make it possible to further process the obtained elements. The range of available materials that can be processed with FDM is constantly growing [9]. Machines for FDM, in comparison with other additive manufacturing technologies, have small dimensions and are easy to maintain. They are also quiet and clean, which makes them available for use in design offices, hospitals and medical facilities [2,3,12].

One of the largest disadvantages of FDM is the relatively low mechanical properties of obtained products (tensile & bending strength, impact resistance, elongation at break and others) [13,14]. Apart from being lower than expected, these properties also often have uncertain, hard-to-predict values and are anisotropic on a macroscopic level [15]. The influence of the manufacturing process parameters on mechanical properties of products made using FDM, and economical coefficients of the process have been thoroughly studied worldwide [13,15,16,17].

One of the most popular classes of 3D-printed medical products in wide use are orthopedic supplies, especially limb orthoses [18,19,20]. The orthoses are medical supplies that keep a selected part of a patient’s body rigid and safe during healing or convalescence. This is usually accomplished by immobilizing and protecting the area around a joint from deformations and physical damage. The orthoses may also be used for enforcement of a specific position and mutual orientation of various body parts [21]. They can be universal, relatively inexpensive orthoses or customized (much better in terms of healing function and comfort) products made for a specific patient based on their anatomic measurement [22].

Wrist-hand orthoses are commonly produced worldwide, as wrist injuries are one of the commonest fractures, specifically around 25% of fractures among the pediatric population and up to 18% in the elderly age group are distal radius fractures [23]. The most simplistic treatment approach is a plaster cast, although it is very uncomfortable for use and cannot be removed without destroying it. It can be replaced with a thermoplastic orthosis, made in a few available sizes or customized for a given patient, which generally brings better results [24].

A typical, traditional process of customized orthoses manufacture consists of manual activities, such as taking a measurement from a patient by making a so-called negative from a plaster cast, then making a positive model out of it, often by manual layering of thermoplastic and other materials. It has many disadvantages, including time and cost. The modern process introduces repeatability, as patient data are gathered and stored digitally, by means of noncontact measurements using standard 3D scanning or medical-grade techniques such as MRI (Magnetic Resonance Imaging) or CT (Computed Tomography). After gathering data, work of a biomedical engineer is required to digitally shape a customized product, maintaining anatomic and technical correctness. Next, it can be manufactured, which is often done rapidly by industrial 3D printing processes [25].

One of the largest problems in 3D printing of customized orthopedic supplies is requirement of specialized engineering knowledge. The patient’s anthropometric data must be gathered and processed, usually manually. This can generate many inaccuracies [26]. Obtaining a shape requires many hours of advanced surface modeling in CAD systems [23]. Additionally, 3D printing of thermoplastic products with satisfying values of accuracy and strength is difficult, as process parameters significantly influence properties of obtained parts [27]. Therefore, traditional processes of making plaster casts have not yet been replaced with 3D printing. Studies on how to make the data gathering, processing and manufacturing easier and more available in general medical practice are regularly carried out [23,24,28]. Automation of certain engineering tasks seems a promising direction [25]. As the authors of a paper [28] note, the lack of dedicated, easy-to-use design software significantly limits the possibility of widespread use of additively manufactured orthoses. They offered their own technical solution, which is so simple and intuitive that it can also be used by medical personnel with no experience with traditional CAD 3D systems for engineers. The authors of the work [27] suggest that the software for the design of orthoses should use only the simplest modeling operations, performed directly on the meshes from the scanning process.

This paper mainly addresses the issue of 3D printing of openwork wrist–hand orthoses, an idea popularized by the design of Jake Evill [29] and described in known literature [19,24]. Currently, there are many designs available, such as the Xkelet [30], Osteoid [31] and many others [24]. However, the current literature does not contain works on selection of the best material and process parameter combinations to obtain optimal results in terms of strength, fit and surface quality, usability and cost and time, especially regarding orthoses that are designed automatically. The results of experimental work on the matter are presented in this paper.

## 2. Materials and Methods

### 2.1. Research Concept and Plan

**Figure 1:**
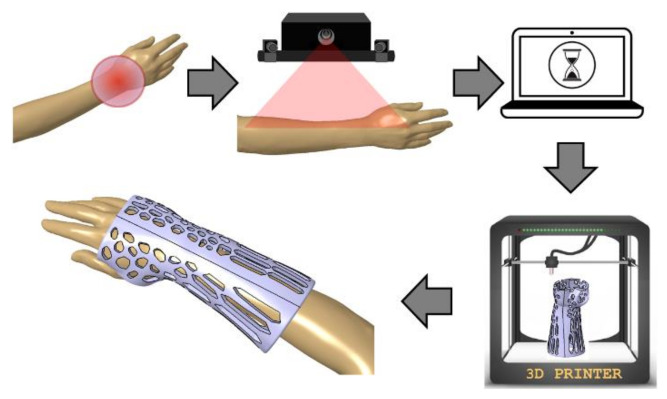
Scheme of work of the proposed AutoMedPrint system.

The research was realized in the scope of the project “Automation of design and rapid manufacturing of individualized orthopedic and prosthetic supplies on the basis of data of anthropometric measurement”. To solve the problems of existing approaches to manufacturing of customized wrist–hand orthoses, an entirely new concept of an automated system is proposed. The system, abbreviated AutoMedPrint (automated 3D printing of medical products), has a task of automated design and production preparation of individualized orthopedic supplies—mainly limb orthoses and upper limb prostheses. The system’s concept is described in the authors’ earlier works [32] and scheme of its operation is shown in Figure 1. The outcome of system operation is design of a new, customized model of an orthosis in less than 15 min, then 3D printing it and delivering to the patient in under 36 h at a cost lower than traditionally made equivalent products.

The main aim of the research work described in the paper is the design and realization of experiments focused on selection of materials and parameters of an additive manufacturing process of the openwork wrist–hand orthosis designed fully automatically based on a 3D scan of a given patient.

### 2.2. Design of a Customized Orthosis

The wrist–hand orthosis was designed in a fully automated way, using an existing intelligent CAD model in the Autodesk Inventor software (version 2019, Autodesk Company, San Rafael, CA, USA). The intelligent model was made by the authors as a part of previous studies [32]. The orthosis consists of two parts (horizontal division) that are fit together by a snap fit shape connection (grooves and splines) and is of openwork, lightweight build. Wall thickness is 4 mm and offset between a patient’s geometry, and the inner surface is 3 mm. The orthosis was designed based on a 3D scan of a right forearm of a 21-year old female patient of 160 cm height and average body weight. This made basic length selection (140 mm) possible.

**Figure 2:**
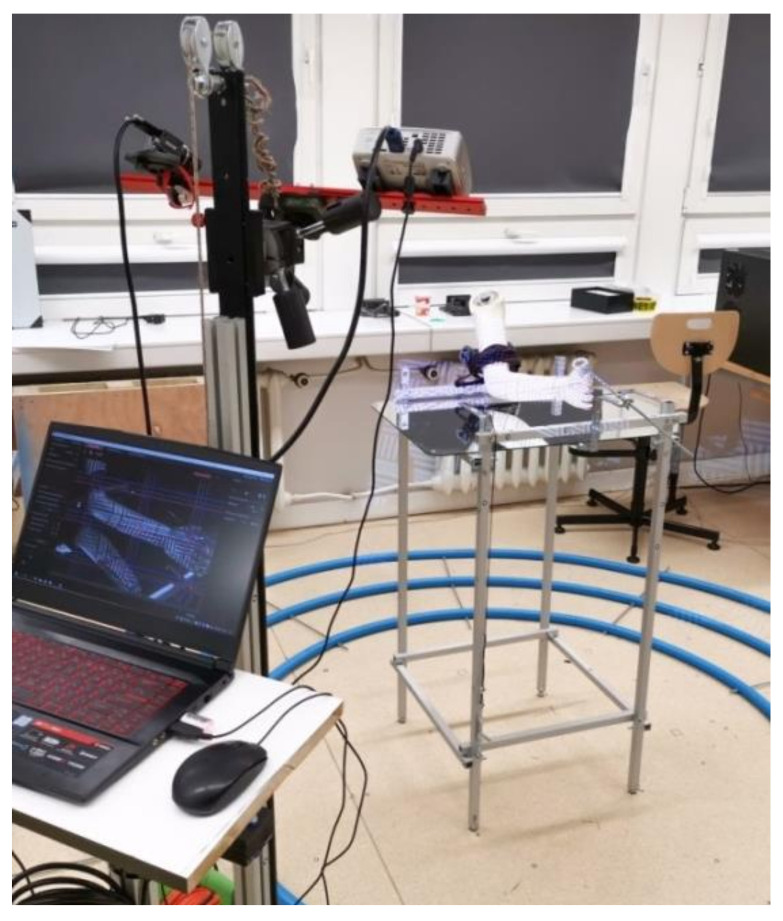
Work stand with a 3D scanner.

**Figure 3:**
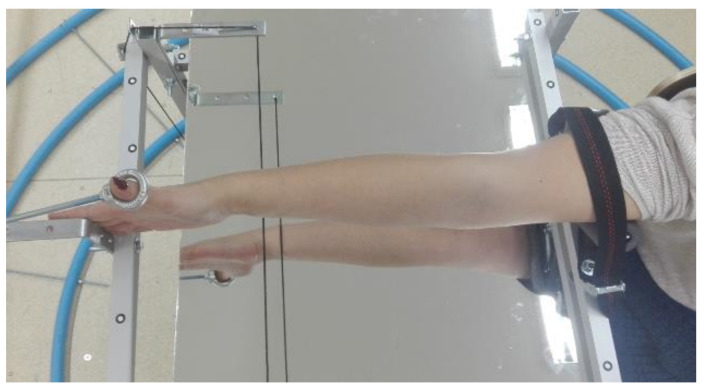
Forearm position during scanning.

First, the patient’s forearm was 3D scanned on a specially designed work stand, equipped with a David SLS-3 optical scanner (David Vision Systems GmbH, Koblenz, Germany) (Figure 2) moving along a circular track. In total, 6 scans were made—4 direct scans in different positions of the scanner and 2 scans of its mirror image. This is a novelty—during preliminary studies it was found that it is possible to capture 3D point clouds of objects visible in a regular mirror using structural light scanning, so there is neither need of changing a patient’s position during the scanning nor changing angles between the scanner and the work stand. During the whole process (lasting approx. 5 min), the patient’s forearm must rest on a special construction and remain still (Figure 3).

**Figure 4:**
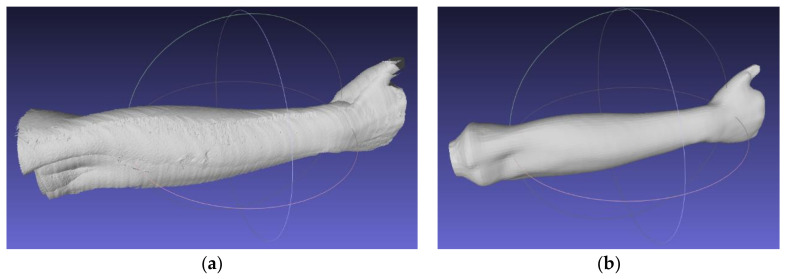
Obtained forearm geometry. (**a**) Raw meshes after merging and cleaning; (**b**) result of screened Poisson surface reconstruction algorithm.

Using open-source MeshLab software (version 2019, Istituto di Scienza e Tecnologie dell’Informazione, Pisa, Italy), an automated algorithm of 3D scanning data processing was created by the authors. The algorithm first, aligns the scans (using transformations obtained in the work stand calibration procedure), then removes the unnecessary data and transforms the scans into a correct global coordinate system. The next stage is to create an entirely new mesh using the screened Poisson surface reconstruction algorithm [33]. The final forearm geometry (mesh) is shown in Figure 4.

The last stage of the automated MeshLab algorithm mentioned above (is to create a series of section planes, which makes it possible to obtain point sets. The section planes are made perpendicularly to the arm axis, each 4 mm. Based on the manually measured length of the whole forearm, wrist and thumb location, automated selection of sections is performed. The intelligent CAD model, mentioned before (a variant template, adjusting its geometry based on specific patient data) requires 11 sets of points. Based on point coordinates, parameterized spline curves are created, which after multi-section extrusion and addition of an offset, create the main shape of the individualized orthosis. The points are initially preselected with an automated algorithm created in VBA language in an Excel spreadsheet.

**Figure 5:**
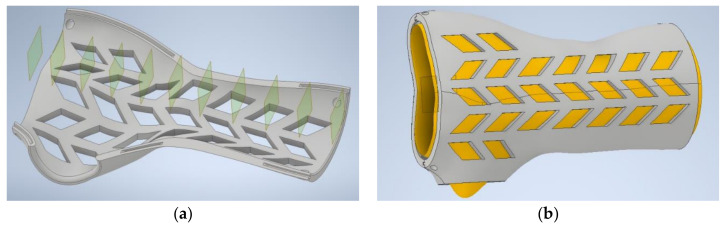
Automatically designed customized orthosis. (**a**) Lower part with visible grooves for assembly, (**b**) complete orthosis, juxtaposed with recreated patient’s forearm geometry.

The result of the automated data processing algorithm is an Excel spreadsheet, and the data are fed to the 3D model in the Inventor software. All operations after the scanning, before the intelligent CAD model is launched (automatically taking data from the spreadsheet and generating an individualized 3D model), are realized fully automatically without operator involvement, thanks to appropriately written macros and scripts. Figure 5 presents a 3D model of the orthosis.

Table 1 contains summary of time of orthosis design process using automated data processing algorithms and an intelligent CAD model.

### 2.3. Manufacturing

The automatically designed individual wrist–hand orthosis was manufactured in several dozens of pieces, with varying materials and process parameters. Destructive and nondestructive testing was performed on the obtained products. For each product, 4 aspects were assessed: manufacturing process (stability, assembly fit), economical aspect (manufacturing time and total cost), accuracy (patient fit, surface quality) and, strength (maximum force recorded at the bending test). Variability of manufacturing parameters was limited to values that were selected in preliminary studies as the most suitable for large-scale testing. The changeable parameters (apart from materials) were product orientation in the working chamber, layer thickness and infill percentage.

The manual post processing of the obtained products was limited to the simplest activities: support removal, assembly and basic manual grinding. Fit, accuracy and strength were assessed after processing.

The manufacturing processes were realized with a Raise 3D Pro machine (Raise 3D Technologies, Inc., Irvine, CA, USA), with a working chamber sized 305 × 305 × 605 mm, with a dual extruder, each with 0.4 mm diameter. Four materials were used: ABS, PLA, nylon (PA12) (Spectrum Group, Pęcice, Poland) and high-impact polystyrene (HIPS) (MakerBot, New York, NY, USA), in the form of 1.75 mm-diameter filaments. Material processing characteristics (based on the material supplier data) are presented in Table 2. Constant material parameters were kept during manufacturing. The temperatures and extrusion speeds were selected after the preliminary tests on simple samples, and the most suitable values recommended by the producer were selected (bringing the most stable process, without layer disjoint, underextrusion or other typical errors). The selected temperatures are shown in Table 2. The extrusion speed was assumed 80 mm/s for all the materials except nylon (40 mm/s).

The orthoses were manufactured in two different orientations: vertical and horizontal. In vertical manufacturing, no support structures were used. In horizontal orientation, the automatically generated supports were used. They were made of the same material as a given part and were later removed by manual breakage.

Three differing strategies were formulated regarding the layer thickness and infill:

- Economic (econo)—15% infill, 0.3 mm layer;
- Accurate (accura)—15% infill, 0.15 mm layer;
- Strong (strong)—90% infill, 0.3 mm layer.

Solid (100%) infill was avoided due to a PLA processing requirement—this material is not suited for manufacturing with such infill density (it generates many volume errors and high internal stress, as can be concluded from authors’ practical experience and preliminary studies). For each unique combination of process parameters (material, orientation, strategy), 2 complete orthoses were manufactured. Each orthosis (consisting of 2 parts) should be treated as a single specimen subjected to fit and strength tests (the results were averaged). Considering 4 materials, 2 orientations and 3 strategies, 24 unique combinations were obtained. Therefore, 48 complete orthoses were manufactured and tested (96 manufacturing processes were performed).

### 2.4. Product Assessment Methodology

As briefly mentioned above, the following aspects were assessed in the obtained products:

- Process coefficients: (a) process stability—how many process instances were stable without operator intervention, on a fully functioning machine without avoidable operator error; (b) part fit—the possibility of assembling parts together without time-consuming processing by machining;
- Economic coefficients: manufacturing time and cost;
- Technical coefficients: strength (force at break in the bending test) and accuracy (fit/no fit, descriptive surface quality assessment, 3D scanning of selected orthoses).

In terms of process coefficients, the process was considered stable when it could be left unsupervised for the whole duration and yield a usable product. Major failures (such as product disjoint from the machine table) are considered as instabilities, while minor errors (such as temporary nozzle clogging, omitting part of one layer or visible droplets of material) were not.

Considering the economic coefficients, real machine work time was measured, as well as real material consumption (each product was weighed, before and after support removal). The cost was calculated similarly to how a commercial order would were priced, including the following components:

- Material consumption: model and support, by weight;
- Manufacturing time: machine work cost per hour was calculated based on market price of the machine, divided by the standard period of consumption of fixed capital (2 years of work, 8 h a day);
- Operator work time: assumed nonzero if post processing was longer than 5 min.

**Figure 6:**
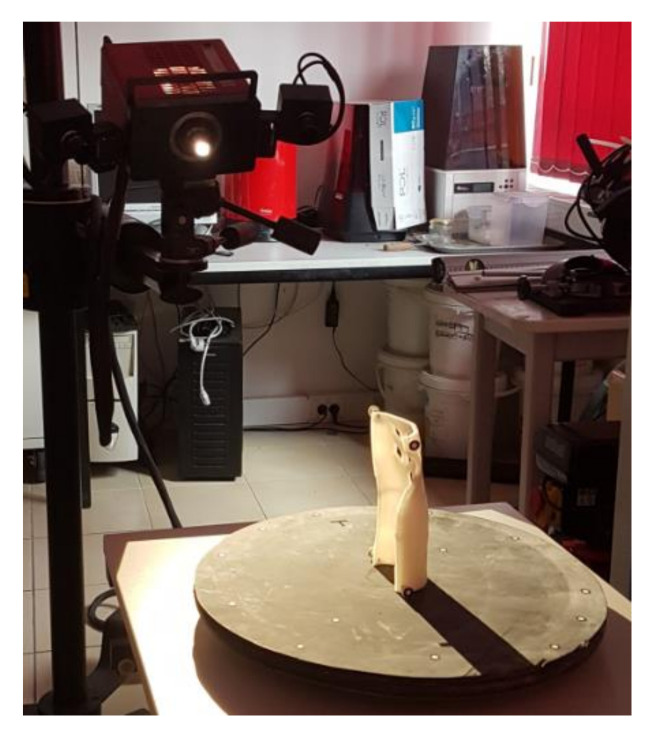
Measurement of an orthosis with a 3D scanner.

The patient fit and accuracy assessment was subjective—the patient wore all the manufactured orthoses and used each for 15 min, assessing them in a binary way (ok–not ok) and made a surface quality assessment on a 1–3 scale. Selected orthoses representing all the materials, orientations and strategies were also subjected for acceptance of a qualified orthopedist. The accuracy was also measured objectively—selected orthoses were 3D scanned using a GOM Atos I structured light scanner (GOM GmbH, Braunschweig, Germany) (Figure 6) and the resulting scans were compared with the nominal CAD model, to find general dimensional deviation (in a standard quality control procedure). The strength tests were performed as a final stage and are described in more detail in the next chapter.

As a final assessment of a given product (an orthosis made of a given material, with a given set of parameters), a decision of acceptance/rejection was made. The rejection criteria were as follows:

- Less than 100% process stability (no 2 successfully produced instances);
- Unfitting parts/non-fit to the patient, more than 1 mm of average dimensional error;
- Too high cost—equal or exceeding a cost of an equivalent commercial product (75 USD assumed as a threshold);
- Too long manufacturing time (longer than two work shifts—16 h, assuming sequential manufacturing of two parts);
- Lack of strength (less than 300 N at the moment of product failure).

### 2.5. Strength Testing Procedure

**Figure 7:**
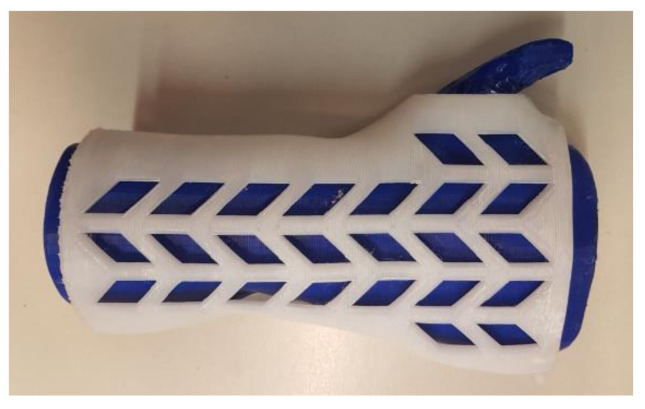
Orthosis on a two-material forearm phantom.

The strength testing of the manufactured wrist–hand orthoses consisted of a destructive quasi 3-point bending test of the whole orthosis. To simulate bending with a patient’s hand inside, a two-material forearm phantom was manufactured. Based on the 3D scan (Figure 4b), an outside shell and a core in the shape of a conical rod were modeled. The shell was made of TPU (elastic thermoplastic polyurethane) (Fiberlab S.A., Brzezie, Poland) material, infill 15%, the core was made of ABS (Spectrum Group, Pęcice, Poland), infill 30%. This is how the imitation human forearm was manufactured. A tested orthosis was assembled on a forearm phantom (Figure 7).

**Figure 8:**
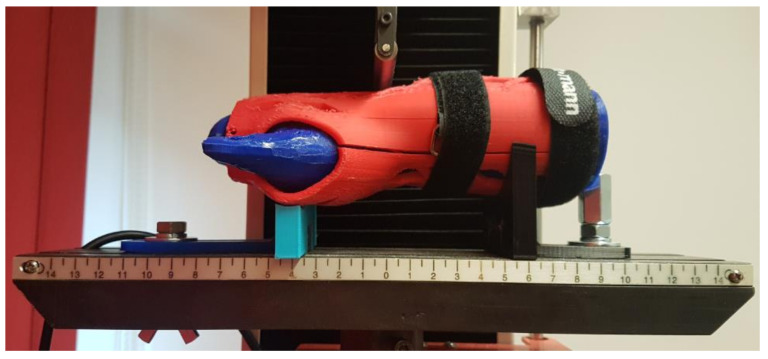
Bending test of an orthosis with a forearm phantom, dedicated supports visible.

The strength tests were performed with the universal strength testing machine Sunpoc WDW-5D-HS (Sunpoc, Guiyang, China). The course of the experiment was developed based on ISO 527-2:2012 standard [34]. The load was placed in the wrist joint, from the upper side. Special shaped supports were designed and manufactured out of ABS material and placed and screwed to the test machine’s rail, 40–50 mm far from the load. During loading, the orthosis was not additionally fixed—the supports significantly limited the freedom of movement of the product. The whole set, immediately before testing, is presented in Figure 8.

The result of each test is a course of a load–displacement diagram, obtained from the used strength testing machine. The test was carried out until an orthosis was destroyed (by cracking) or visibly deformed. As a threshold of positive evaluation of a given orthosis, a load of 300 N was assumed (representing a static bending using an item of 30 kg mass). The value was set arbitrarily, based on discussion in the project team, with mechanical and biomedical engineers and with expert participation of orthopedists and physiotherapists, who stated that in practice, such orthoses do not need to bear higher loads.

## 3. Results

### 3.1. Manufacturing Results and Economical Coefficients

The manufacturing was realized according to the plan and obtained a complete set of orthoses. No major process stability problems occurred. Of the several problems that were noticed, most were a result of operator error (wrongly calibrated or improperly cleaned worktable, clogged extrusion head). In the case of an unstable process when instability source was detected as human error, the process was repeated. All the other problems were minor, which means they did not cause failure in manufacturing of a given part. The common properties of all the occurring stability problems are noted below:

- All occurred when orthoses were manufactured vertically;
- None occurred in the econo strategy;
- None occurred with the ABS material, most occurred with nylon and HIPS (few with PLA);
- Observed problems were mainly disjoin from the table and machine errors (layer translocation, material blockage, material droplets in unwanted places, etc.).

Altogether, of the realized processes, 8 stability problems not resulting due to human error were recorded (mainly partial table disjoin and layer translocation). The problems that could potentially (with more repetitions) lead to the product manufacturing fail occurred only with PLA and nylon in the strong strategy and could be eliminated by making table surface more adhesive and by reducing the extrusion speed. The “safest” combination is ABS material with the orthosis manufactured horizontally in the econo strategy. No major difference was noted for two parts of the orthosis.

It can be assumed that in general, given the set of applied process parameters, acceptable stability was achieved—the process does not require an operator’s supervision (which is relevant from the viewpoint of the whole system, which should work in a fully automated way).

**Figure 9:**
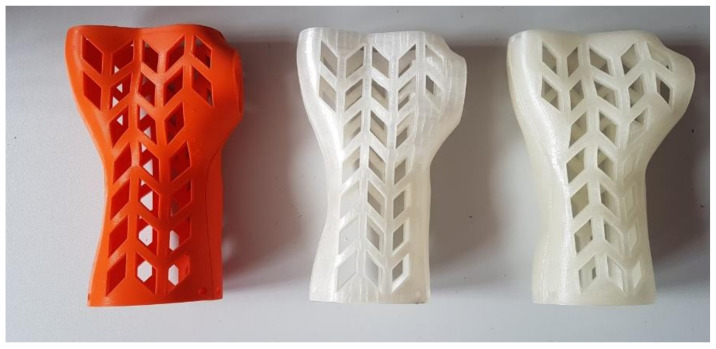
Orthoses manufactured vertically from PLA, strategies. (**left** to **right**) Econo, accura, strong.

**Figure 10:**
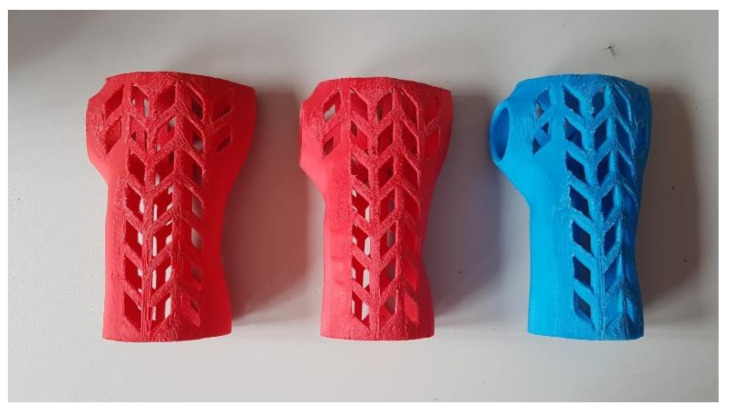
Orthoses manufactured horizontally from ABS, strategies. (**left** to **right**) Econo, accura, strong.

**Figure 11:**
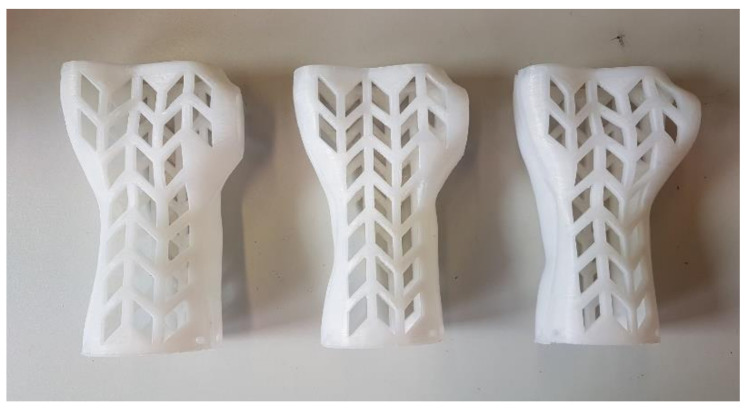
Orthoses manufactured vertically from nylon, strategies. (**left** to **right**) Econo, accura, strong.

Figure 9, Figure 10 and Figure 11 present examples of manufactured orthoses, of various materials and process parameters.

The manufactured orthoses were generally very lightweight. The upper part of the orthosis weighed in between 19 and 39 g, while the lower part in between 31 and 61 g. In total, no complete orthosis exceeded weight of 100 g, most of orthoses weighed around 60–70 g, with the lowest recorded value of 53 g. As expected, the heaviest orthoses are manufactured in the strong strategy out of PLA (the densest material), while the lightest—of ABS, in econo or accura strategy. The only two influential factors here are the infill (strong versus two other strategies) and material density, orientation and layer thickness having no statistical impact on the total weight.

**Figure 12:**
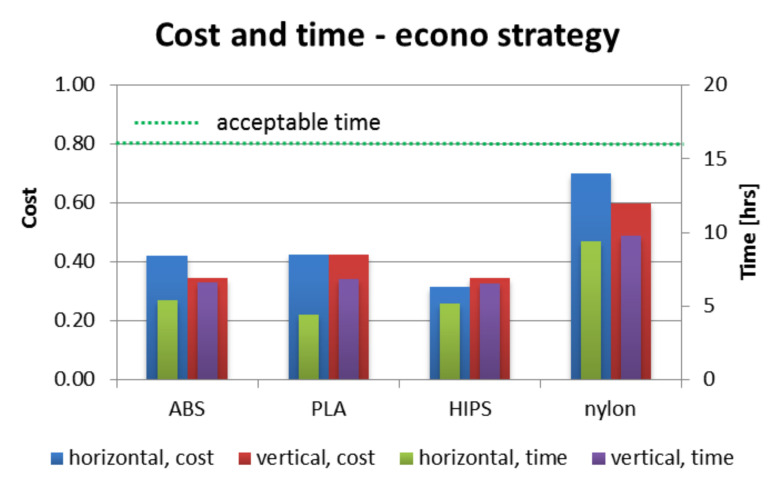
Cost and time of manufacturing—econo strategy.

**Figure 13:**
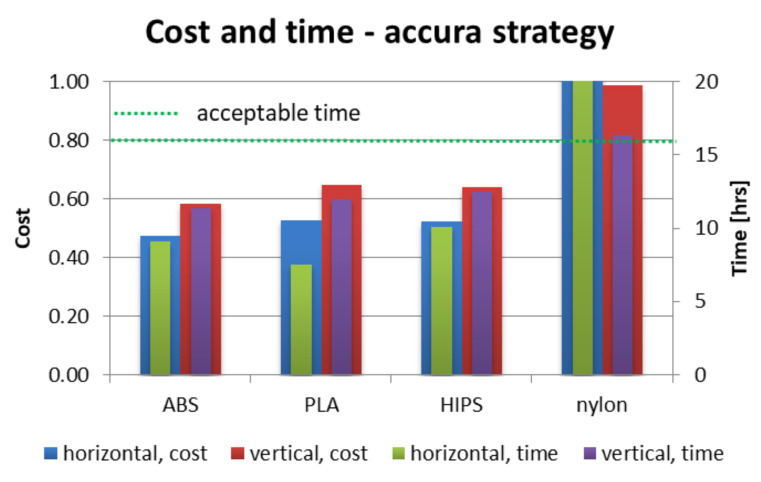
Cost and time of manufacturing—accura strategy.

**Figure 14:**
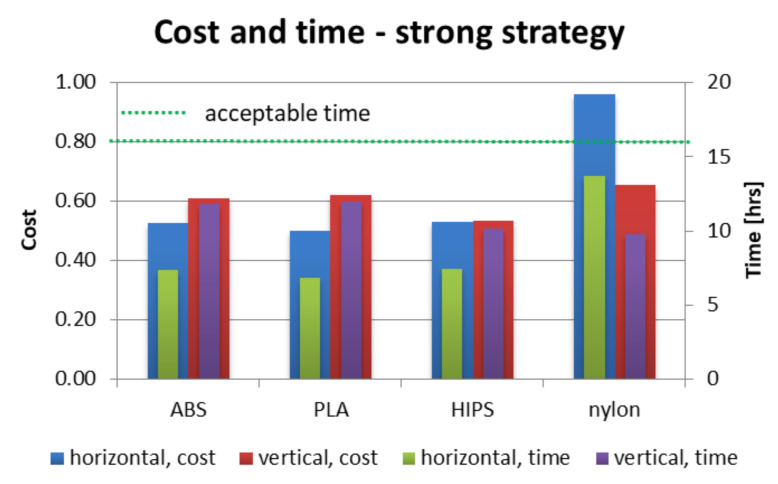
Cost and time of manufacturing—strong strategy.

Figure 12, Figure 13 and Figure 14 present juxtaposition of costs and times of manufacturing particular orthoses. The price was scaled in a way that 1.0 value is a maximal acceptable price, equal to a market price of a mass-produced (non 3D-printed) orthosis, available in medical shops—this price was 75 USD on the local market at the time of conducting the studies. An acceptable time was assumed as 16 h, which is two work shifts. This is enough time (in perfect conditions) to supply a patient with an orthosis on the second day after scanning, or even the same day, if both parts are produced simultaneously (on two machines).

As shown in Figure 12, Figure 13 and Figure 14, nearly all process parameter combinations make production of affordable orthoses possible. They can be manufactured cheaper than the equivalent non-individual, mass-produced orthoses. The one case where this is not true is in a nylon orthosis manufactured in accura and strong strategies in horizontal orientation—this is due to a large amount of support material and low extrusion speed. Most orthoses also fulfill the short time criterion—only the nylon orthosis in the accura strategy exceed the acceptable time, with the horizontally manufactured one being the slowest to manufacture (and the most expensive) by a wide margin—it took almost 28 h to obtain both parts of the orthosis, with the cost coefficient of approx. 1.8 (not exceeding 1 for all the other combinations).

### 3.2. Accuracy and Fit Results

For all the manufactured orthoses, it was possible to fit together two parts of the orthosis immediately after manufacturing (and, optionally, support removal), except for the nylon orthoses—for those, no orthosis could be put together until slight manual grinding procedure was performed (which also slightly increased the predicted cost of selected orthoses).

All the manufactured and assembled orthoses were tested by the patient and all were fit—none were rejected on that ground. All the selected, representative orthoses were also accepted by the orthopedist, as suitable for wrist bone fracture treatment.

In terms of surface quality and accuracy of some orthoses, there were critical remarks. The orthoses, for which the “1” surface quality score (in 1–3 scale) was assessed were all the orthoses manufactured in horizontal orientation (material and strategy were of no matter here). On the other hand, the “3” score was awarded to nearly all the orthoses manufactured vertically, excluding nylon orthoses manufactured with the econo strategy. In the descriptive evaluation, better esthetics and surface quality of accura orthoses was underlined; however, it was purely a subjective assessment. The infill percentage had no impact on this assessment—strong and econo orthoses were awarded the same scores. This confirms that the orientation is the most important factor influencing the accuracy and visual quality, with the layer thickness being the secondary factor and infill having no significant importance.

**Figure 15:**
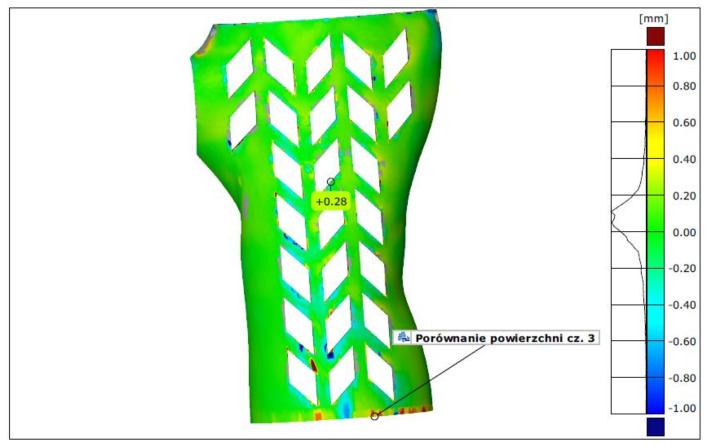
Exemplary result of accuracy examination, PLA, accura strategy and vertical orientation.

In terms of measurement by 3D scanning, the lowest average deviation (the mean fit error) was found for orthoses made of PLA in vertical orientation—it was between 0.13 and 0.18 mm. The largest deviations were found, as expected, in the horizontally manufactured orthoses in the econo and strong strategies (various materials)—they ranged from 0.43 mm to 0.57 mm. The highest deviation for orthoses manufactured using the accura strategy did not exceed 0.28 mm, which translated into the lowest average for this group (0.2 mm). The differences between the econo and strong strategies are not statistically significant. As no orthosis presented deviation higher than the assumed 1 mm, none was marked as unacceptable on these grounds. An example of a colorful deviation map, resulting from comparison of a 3D-scanned orthosis with the nominal CAD model is shown in Figure 15.

### 3.3. Strength Testing Results

**Figure 16:**
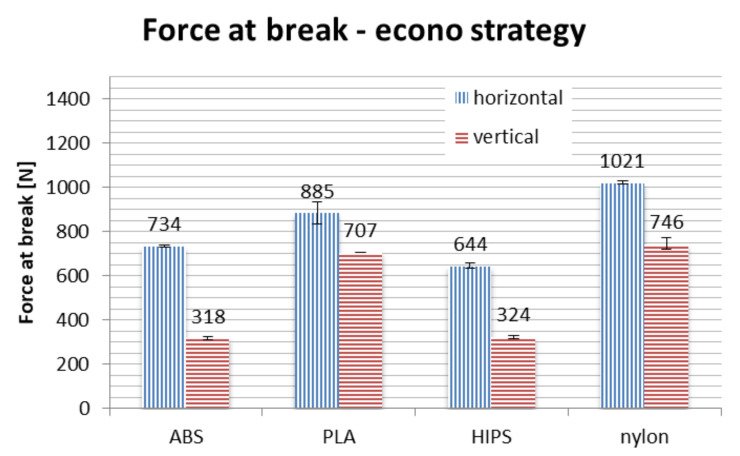
Strength of manufactured orthoses—econo strategy.

**Figure 17:**
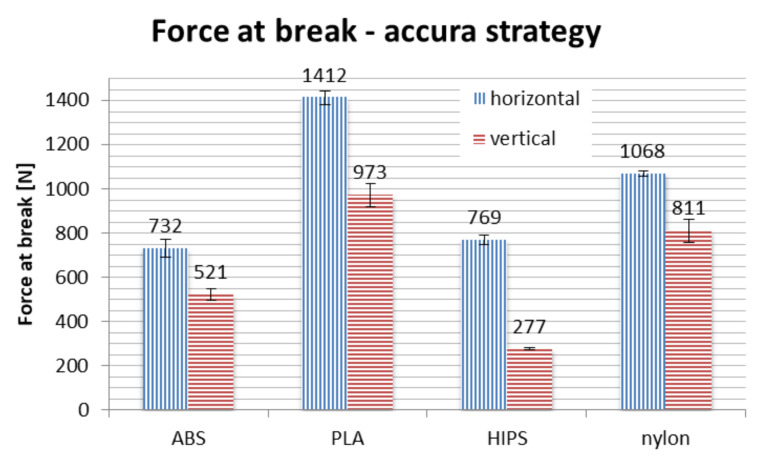
Strength of manufactured orthoses—accura strategy.

**Figure 18:**
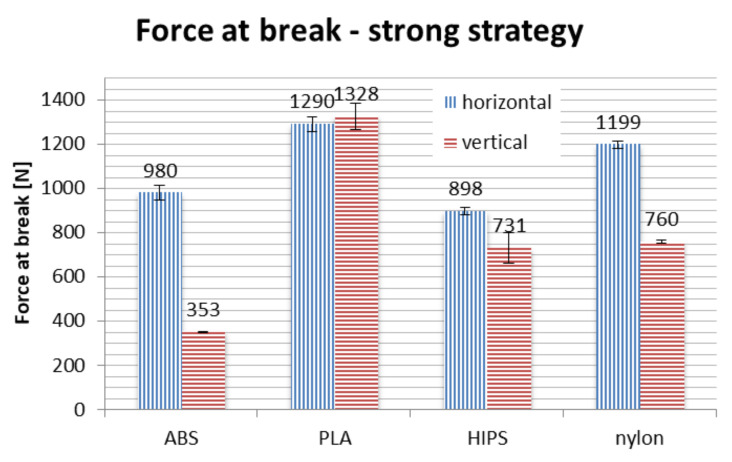
Strength of manufactured orthoses—strong strategy.

Results of strength testing divided by strategies, for different materials and orientations, are shown in Figure 16, Figure 17 and Figure 18 and in Table 3.

In terms of material, PLA is the strongest if taken on average. No process parameters combination brings a result of force at break lower than 700 N, with average of approx. 1000 N. The nylon is the close second, with average value of above 900 N, no orthosis damaged below approx. 750 N. The ABS and HIPS are very close to each other in terms of raw value comparison.

**Figure 19:**
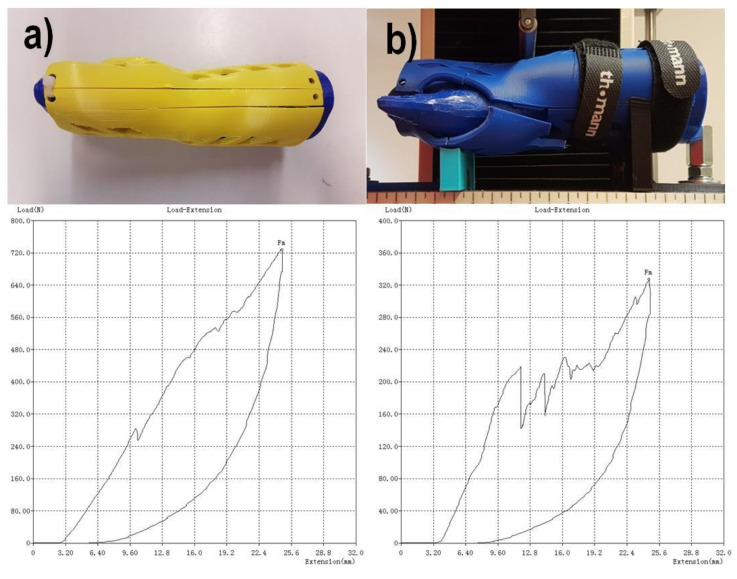
Different mechanisms of failure (ABS, econo), (**a**)—horizontal orientation, (**b**)—vertical orientation, juxtaposed with force–displacement diagrams (below the photographs).

However, detailed comparison brings interesting results. The orthoses made of ABS lose approx. 50% of their strength when manufactured vertically, rather than horizontally. The only other material that shows such a difference between orientations is HIPS. This can be attributed to low strength of adhesion between layers in orthoses made of these materials, as the failure in vertical orientation was always due to layer disjoint (a corresponding phenomenon in monolithic products is brittle cracking), as shown in Figure 19.

The PLA material also loses a part of its strength when the orthosis is manufactured vertically, which is most visible in the accura strategy (lower layer thickness). The loss of strength for nylon is relatively stable for all the orientations, even taking into consideration the raw numbers—statistically, strategy is insignificant for this material, the layer thickness and infill percentage seem to have no impact on the recorded strength in the performed test.

Orthoses manufactured in different orientations also have different mechanisms of failure (locations and propagation directions of fractures), as shown in Figure 19. This is due to directionality of the layers and lower strength of layer bonds versus the material internal strength. The ABS and PLA are similar in this aspect, while nylon behaves differently—the fractures are barely visible, and the orthoses usually keep their integrity. Therefore, maximum values of strength obtained for nylon should be treated slightly differently—the bending force at which nylon orthoses lose their usability (due to falling apart in pieces) is probably much higher than any orthoses of PLA/ABS.

Bending tests of orthoses manufactured in the strong strategy brought unpredictable results. In general, on average, use of this strategy increases the recorded maximum force values. However, two counter-intuitive occurrences were found: the strength of vertically made PLA orthoses is greater than those made horizontally, while orthoses made of ABS material in vertical orientation are significantly weaker than could be anticipated, with values comparable to the econo strategy.

## 4. Discussion

The main goal of the performed experiments was to prove it is possible to manufacture wrist–hand orthoses of good quality (both strength and fit/surface quality) by 3D printing with acceptable time and cost and with no process supervision. This goal was generally demonstrated, as visible in the values of strength tests and economic coefficients, as well as considering the fit assessment. The secondary goal was to select the strategies, materials and orientations to be used in the final version of the automated design and manufacturing system, which is being developed by the authors (AutoMedPrint). Each aspect—strategy, material and orientation—will be discussed separately.

The strong strategy did not bring significant beneficial results in terms of recorded maximum strength. This was probably due to the orthosis itself being thin-walled and thus not getting a limited strength bonus when the infill was increased. Moreover, results for vertically made ABS and PLA orthoses are unpredictable, probably caused by internal stress introduced by tight infill packing. Considering all this, as well as the noted process stability problems, it was decided to dump this strategy and not recommend it in the final version of the system. It does not bring any real benefit, while the economic coefficients of obtained orthoses are unfavorable. The orthoses manufactured at the econo strategy should be acceptable for most users. However, the accura strategy makes possible an increase of accuracy, surface quality and strength at a significantly increased cost. The choice of which strategy to use will be left to the users of the system, econo being the default choice.

In terms of materials, with all results considered and material characteristics not taken into account in the performed study, it was decided to recommend three of four tested materials for use in the final version of the automated design and manufacturing system. These materials are PLA, ABS and nylon—HIPS was left out due to unfavorable test results, poor printability and general properties. When the strong strategy is not considered, HIPS is the weakest material overall, with certain problems with process stability, known brittleness and low chemical resistance.

PLA presents the highest strength at break recorded in the tests. It is also cheap and possible to process on virtually any fused deposition modeling/fused filament fabrication printer. It is also organic, thus environmentally and skin friendly. The accuracy of measurement makes it possible to observe the lowest mean deviations for orthoses of this material. However, in full view of its properties, it cannot be the only recommended material for manufacturing wrist–hand orthoses. It has low temperature resistance—in practice, any temperature above 50–60 °C (such temperatures are common, e.g., in car interiors in summer all over the world) can soften it, making the product fragile and lose its anatomic shape. It also has lower chemical resistance than the other materials and is more prone to breaking on impact (impact tests were not done yet—it is planned in the future).

The ABS material—although not the best in the presented tests (both in terms of accuracy and strength)—itself has higher impact resistance than PLA and is generally considered tougher by available literature [35]. It is resistant to any temperature that may be encountered by the user of the orthosis in day-to-day use. The bending test results are acceptable for this material, as all the orthoses were above the assumed threshold of 300 N. ABS is neither skin friendly (must be sterilized before and during use), nor environmentally friendly. Its cost and processing capabilities are similar to PLA (a slightly smaller number of 3D printers are able to process ABS).

Nylon is a material which is considerably more expensive and harder to manufacture than the other two. However, it has elastic properties and is very resistant to both temperature and chemical agents. Its bending strength (regarding values obtained in tests) is almost independent on strategy and in itself very high. The material also has high impact and scratch resistance. As mentioned above, the bending force at which nylon orthoses lose their usability is probably much higher than of any other orthoses, as the recorded values were only the maximal values. For the other materials, the maximum force indicated a moment of failure, usually related to a major and visible fracture. For nylon, it marked a moment of transitioning into a plastic state, after which the orthoses kept deforming without breaking—this could be useful in certain applications, e.g., bearing dynamic loads. This alone is a reason for keeping the nylon as a possible recommended material for wrist–hand orthoses, as in the accuracy and fit tests the manufactured orthoses got worse results. Nylon is mildly environmentally friendly, has certain esthetic values and is friendly to skin.

Taking all of this into account, it was decided to recommend regular users to select the PLA material if they do not predict the orthosis suffering dynamic loads or higher temperatures—in such case, ABS is recommended. If the orthosis must bear higher loads, be temperature-resistant, partially elastic and skin friendly, nylon is recommended, at higher costs.

Considering strength only, horizontal orientation is the best. However, high accuracy and surface quality are not possible with horizontal orientation. As it is the parameter that has the highest influence on all technical and economical coefficients and neither of the two orientations can be determined as definitely better than the other, the decision should be left to the user (patient advised by orthopedist). However, the default option is vertical orientation, as all the orthoses of recommended materials and strategies meet the strength criteria and are also smoother and better rated by the users than the orthoses made in horizontal orientation.

In the final version of the automated system, the user will have a choice regarding the material and process parameters. The default recommended choice will be a PLA material, econo strategy (15% infill, 0.3 mm layer thickness) and vertical orientation for better look and feel of the product. If the price will be unimportant, the accura strategy should be selected instead, in vertical orientation for even better accuracy and surface quality or in horizontal orientation for better strength.

## 5. Conclusions

All research was conducted as planned and the results are mostly compliant with initial expectations of the researchers. The unexpected behaviors of orthoses 3D printed with almost monolithic infill (strong strategy, 95% infill) are worthy of further study and analysis. More samples of ABS and PLA should be produced on different machines to ensure this high discrepancy of intuitive predictions and experimental results is not a result of repeating machine error or improper selection of a different parameter (such secondary parameters are, example given, retraction distance, platform temperature, extrusion speed and many others) or even an imperfection in the material itself. It is difficult to predict the full properties of such a product, as it is a thin-walled, openwork construction. The authors simultaneously conducted a study on finite element analysis of 3D-printed orthoses. The results of this study will be released as soon as possible, with a general aim of obtaining a robust method of prediction of behavior of 3D-printed orthopedic supplies under load.

The results of the studies have proved that it is possible to obtain a usable and cheap 3D-printed openwork hand orthosis in less than one workday, from measurement to a ready product, with minimal involvement of a human operator and minimal competences required to perform the whole process. The proposed approach for manufacturing orthopedic supplies is reliable to use in hospitals, doctor offices and other medical facilities, with no full-time involvement of an engineer or technician. The 3D printing itself may be realized as an external service.

The studies also proved that PLA is a material that can be considered for making usable medical products by 3D printing and maintaining environmental friendliness. This is especially important in the case of orthoses for treatment of fractures, which are used for several weeks and then disposed of. Easy, eco-friendly disposal of PLA products is another reliable option.

**Figure 20:**
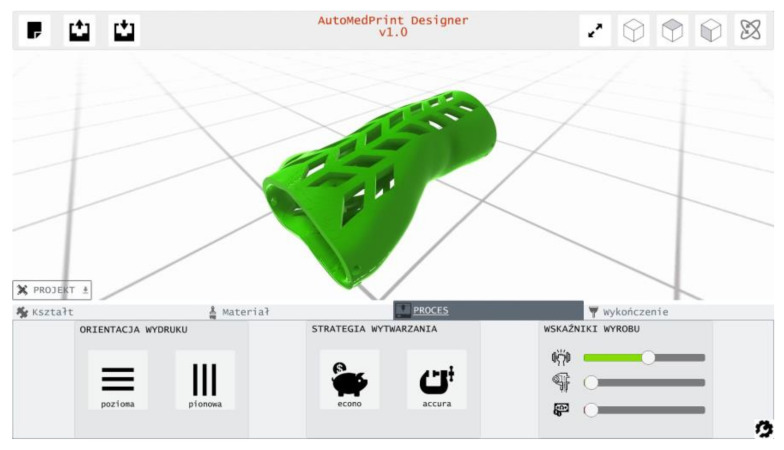
Product configurator—part of the AutoMedPrint system, user interface for the strategy and orientation selection visible below the orthosis model.

The study results and conclusions were considered in the next phase of the project. The product configurator application was built and implemented as a part of the created prototype of the AutoMedPrint system. The selection of materials and process parameters was included as a configurable option, with selection having impact on product looks and properties. The product configurator is shown in Figure 20.

Future studies planned in the scope of the project are experimental testing of more materials (including PETG, PLA–carbon composite and PC) and clinical tests with engagement of real patients suffering a bone fracture.

## Figures and Tables

**Table 1:** Summary of times of orthosis design.

No.	Stage	Time [min]	Time of Human Involvement [min]
1	Measurement (3D scanning)	5 *	5 *
2	Scan assembly	5	–
3	Cleaning, reconstruction	3	–
4	Sectioning and point selection	7	–
5	Automated CAD design	10	–
–	Total	30	5

*—depending on operator skills and patient cooperation.

**Table 2:** Characteristics of materials used.

No.	Name	Properties
1	ABS—acrylonitrile–butadiene–styrene	Density: 1.04 g/cm^3^ Extrusion temperature: 230–240 °C (230 °C) Build platform temperature: 90–100 °C (100 °C)
2	PLA—polylactic acid	Density: 1.24 g/cm^3^ Extrusion temperature: 185–215 °C (210 °C) Build platform temperature: 0–45 °C (45 °C)
3	HIPS—high-impact polystyrene	Density: 1.05 g/cm^3^ Extrusion temperature: 230–255 °C (245 °C) Build platform temperature: 80–100 °C (90 °C)
4	PA12—nylon—polyamide 12	Density: 1.15 g/cm^3^ Extrusion temperature: 240–260 °C (260 °C) Build platform temperature: 80–100 °C (100 °C)

**Table 3:** Summary of strength tests—force at break for various process combinations.

Strategy	Orientation/Material	ABS (N)	PLA (N)	HIPS (N)	Nylon (N)
Econo	horizontal	734	885	644	1021
	vertical	318	707	324	746
Accura	horizontal	732	1412	769	1068
	vertical	521	973	277	811
Strong	horizontal	980	1290	898	1199
	vertical	353	1328	731	760
